# Exploring histone acetylation in ischemic stroke: CREBBP and CKAP4 as candidate biomarkers linked to histone acetylation networks

**DOI:** 10.3389/fphar.2026.1727813

**Published:** 2026-04-02

**Authors:** Yanni Wang, Fan Yang, Jiaojiao Tan, Zhentao Guo

**Affiliations:** 1 Department of Rheumatology, Qilu Hospital (Qingdao), Cheeloo College of Medicine, Shandong University, Qingdao, Shandong, China; 2 School of Medicine, Qingdao Huanghai University, Qingdao, Shandong, China; 3 Department of Neurosurgery, Qilu Hospital (Qingdao), Cheeloo College of Medicine, Shandong University, Qingdao, Shandong, China

**Keywords:** bioinformatics, biomarkers, histone acetylation regulatory genes, immune infiltration, ischemic stroke

## Abstract

**Background:**

Ischemic stroke (IS) is a severe cerebrovascular disorder. Histone acetylation is a key epigenetic modification that is markedly increased and closely associated with enhanced neuronal tolerance to ischemia. Therefore, identifying biomarkers involved in regulating histone acetylation is critical for elucidating the pathological mechanisms of IS and for developing novel diagnostic and therapeutic strategies.

**Methods:**

Transcriptomic data from patients with IS were analyzed to systematically identify the diagnostic biomarkers associated with histone acetylation regulation through an integrative framework combining differential expression analysis, weighted gene co-expression network analysis, multiple machine-learning algorithms, and receiver operating characteristic analysis. Furthermore, the potential biological functions and immune relevance were explored using bioinformatics approaches, including functional enrichment analysis, immune microenvironment evaluation, and disease association analysis. The expression levels of the candidate biomarkers were subsequently validated by reverse transcription quantitative polymerase chain reaction (RT-qPCR) in clinical blood samples.

**Results:**

A total of 44 histone-acetylation-related differentially expressed genes were identified, among which *CREBBP* and *CKAP4* were confirmed as the key biomarkers in IS. Both genes were enriched in the immune-related pathways, including the complement and coagulation cascades. *CREBBP* expression showed a negative correlation with CD8^+^ T-cell infiltration, whereas *CKAP4* expression was positively associated with M0 macrophage abundance. RT-qPCR analysis confirmed the upregulated expression of these genes in IS samples.

**Conclusion:**

*CREBBP* and *CKAP4* were identified as novel candidate biomarkers whose expression patterns are associated with the histone acetylation regulatory gene networks in IS. These findings highlight the potential involvement of these genes in IS pathogenesis and provide new insights for the development of targeted therapeutic and immunomodulatory strategies.

## 1 Introduction

Stroke is the second most common cause of death worldwide that imposes a substantial burden in terms of morbidity, mortality, disability, and long-term socioeconomic impact (Feigin et al., 2021). In 2020, the annual incidence of stroke among individuals aged ≥40 years in China reached 3.4 million, which brought the total number of prevalent cases to 17.8 million and number of related deaths to 2.3 million, creating a significant health and economic burden (Tu et al., 2023). Stroke can be broadly classified into ischemic and hemorrhagic subtypes, of which acute ischemic stroke (AIS) accounts for approximately 87% of all cases. However, the therapeutic options for stroke remain limited, particularly owing to the narrow treatment time window and restricted availability of effective pharmacological interventions (Herpich and Rincon, 2020; Rabinstein, 2020). Currently, intravenous tissue plasminogen activator and emergency endovascular thrombectomy are the main treatment strategies for ischemic stroke (IS), but their clinical outcomes remain suboptimal. Hence, elucidating the pathogenesis of IS and identifying reliable biomarkers are critical steps for improving the early diagnosis, prevention measures, and therapeutic interventions.

Histone acetylation is an epigenetic modification involving the addition of acetyl groups to the lysine residues on histones via histone acetyltransferases (HATs) or removing the acetyl groups using histone deacetylases (HDACs). There is accumulating evidence that histone acetylation regulates the expression of numerous genes involved in diverse biological processes. Acetylation relaxes the chromatin structure to increase accessibility to the transcription factors as well as promote gene transcription and protein synthesis. In contrast, histone deacetylation induces chromatin condensation, resulting in transcriptional repression and reduced protein synthesis (Wang et al., 2009; Schweizer et al., 2013). Previous studies have shown that histone acetylation promotes the activation of genes involved in tissue repair and axonal regeneration while elevated levels of acetylated proteins are associated with increased neuronal resistance to ischemic injury, thereby influencing functional recovery after stroke (Uzdensky and Demyanenko, 2021). In addition, HDAC3 has been reported to attenuate neuroinflammation and ischemia–reperfusion (I/R)-induced brain injury through regulation of the cGAS–STING pathway, highlighting a potential therapeutic target for IS (Liao et al., 2020). Thus, identifying the biomarkers associated with histone acetylation may provide new opportunities for targeted therapeutic strategies against IS.

The present study was aimed at identifying histone-acetylation-related biomarkers in IS using integrated bioinformatics analyses to elucidate the underlying molecular mechanisms and explore potential therapeutic targets. Furthermore, we performed receiver operating characteristics (ROC) analysis to evaluate the diagnostic performances of the signature genes. Lastly, the relationships between these genes and immune cell infiltration were assessed systematically to provide further insights into potential immunomodulatory strategies for IS.

## 2 Materials and methods

### 2.1 Data acquisition

Two IS datasets were retrieved from the Gene Expression Omnibus (GEO) database (https://www.ncbi.nlm.nih.gov/geo/), namely, GSE16561 and GSE202518, which contain clinical information and the corresponding gene expression profiles. The GSE16561 dataset (platform: GPL6883) containing 39 normal and 24 IS blood samples was used as the training set; the GSE202518 dataset (platform: GPL24676) comprising four normal and 12 IS blood samples served as the validation set. A total of 83 histone acetylation regulatory genes (HARGs) were collected from earlier research (Fang et al., 2022).

### 2.2 Analysis of differential genes

The differentially expressed genes (DEGs) between the normal and IS groups were identified using the “limma” package (version 3.52.4) (Ritchie et al., 2015) with the criteria adjusted *p* < 0.05 and |log_2_ (fold change)| ≥ 0.5 (Hao et al., 2025). Then, volcano plots were generated using the “ggplot2” package (version 3.3.6) (Wu et al., 2021). The top-20 upregulated and top-20 downregulated DEGs were visualized using heatmaps generated with the “ComplexHeatmap” (version 2.18.0) (Gu et al., 2016) and “heatmap3” (version 1.1.9) (Zhao et al., 2014) packages.

### 2.3 Weighted gene co-expression network analysis (WGCNA)

The single-sample gene-set enrichment analysis (ssGSEA) scores of the HARGs were used as the trait data for the WGCNA using the “WGCNA” package (version 1.70-3) (Rey et al., 2021). Sample clustering was first performed to ensure data quality and remove the outlier samples. The optimal soft-thresholding power was determined based on the criterion of a scale-free topology fit index (R^2^) > 0.9 (Yu et al., 2025). The gene similarity was calculated from the expression patterns, and a dendrogram was constructed after hierarchical clustering. The gene modules were identified using a dynamic tree-cutting algorithm; modules that were most strongly associated with the HARG ssGSEA scores were defined as key modules, and genes meeting the thresholds |GS| ≥ 0.2 and |MM| ≥ 0.8 were selected for subsequent analyses (Zhang et al., 2024).

### 2.4 Gene enrichment analysis and protein–protein interaction (PPI) network

The histone-acetylation-related DEGs (HAR-DEGs) were obtained from the intersection between the key module genes and DEGs.

Functional enrichment analysis of the HAR-DEGs was performed using the “clusterProfiler” package (version 4.7.1) (Yu et al., 2012) for the Gene Ontology (GO) and Kyoto Encyclopedia of Genes and Genomes (KEGG) pathways with adjusted *p* < 0.05 (Li et al., 2018). In addition, a PPI network was constructed using the STRING database to explore interactions among the HAR-DEGs (Chen et al., 2019).

### 2.5 Machine-learning methods

To identify the key HAR-DEGs in the training set, three machine-learning approaches were applied: support vector machine recursive feature elimination (SVM-RFE) using the “e1071” package (version 1.7-11) (Wu et al., 2022); least-absolute shrinkage and selection operator (LASSO) regression using the “glmnet” package (version 4.1.8) (Kang et al., 2021); Boruta algorithm (version 8.0.0) (Yue et al., 2022). LASSO was performed with 10-fold cross-validation, and the genes were selected on the basis of the lambda.min value. In the Boruta analysis, the parameter mcAdj = TRUE was applied to enable multiple testing correction, and the *p*-values for the feature importance were adjusted using the Benjamini–Hochberg (BH) method. The candidate genes were identified from the intersection of the results obtained from the three algorithms.

### 2.6 ROC analysis

The diagnostic performances of the candidate genes were evaluated using ROC curve analysis with the “pROC” package (version 1.18.5) (Robin et al., 2011). The GSE202518 dataset was used as an independent validation set to further assess the diagnostic accuracy. Genes with significant diagnostic values (area under the curve (AUC) > 0.7; Gong et al., 2025) and consistent expression trends in both the training and validation sets were defined as the histone-acetylation-related biomarkers.

### 2.7 Immune infiltration analysis

Immune cell infiltration was estimated using the CIBERSORT algorithm to quantify the relative proportions of 22 types of immune cells. Differences in immune cell infiltration between the two groups were analyzed using the Wilcoxon test to identify the differentially abundant immune cells. Then, Spearman correlation analysis was performed to assess associations between the biomarkers and enrichment scores of the 22 immune cell types as well as between the biomarkers and differentially infiltrated immune cells (|cor| > 0.3, *p* < 0.05) (Liu L. et al., 2025). To further validate the reliability of the identified immune alterations, the “xCell” package (version 1.1.0) (Aran, 2020) was used to estimate the scores of 64 immune and stromal cell types in each sample from the GSE16561 dataset. Differences in the cell scores between the IS and control samples were then compared using the Wilcoxon test (*p* < 0.05) (Liu K. et al., 2022).

### 2.8 Gene set enrichment analysis (GSEA)

GSEA was performed using the “clusterProfiler” package based on the C2 KEGG gene sets to explore potential biological pathways associated with the identified biomarkers (adjusted *p* < 0.05) (Yuan et al., 2025). Then, correlation coefficients were calculated between the biomarker expression and expression levels of all genes for use as ranking metrics.

### 2.9 Construction of the lncRNA–miRNA–mRNA network

The miRDB (http://mirdb.org) and TargetScan (http://www.targetscan.org) databases were used to predict the miRNAs targeting the identified biomarkers, and the key miRNAs were determined from the intersection of the results from the two databases. The starBase database was subsequently used to predict the lncRNAs associated with these key miRNAs (pancancerNum ≥ 10) (Zheng et al., 2021). An lncRNA–miRNA–mRNA regulatory network was then constructed and visualized using Cytoscape software (Liu et al., 2020).

### 2.10 Prediction of drug–target interactions

To identify potential therapeutic agents targeting the identified biomarkers, we performed drug–target interaction analysis using the Drug Signatures Database (DSigDB; http://tanlab.ucdenver.edu/DSigDB) that integrates drug-induced gene expression signatures and curated drug–target relationships. Interactions with an adjusted *p*-value (false discovery rate) < 0.05 were considered significant (Zuo et al., 2016). The resulting drug–biomarker interaction network was visualized using Cytoscape software (version 3.10.2).

### 2.11 Biomarker expression validation

To validate the transcriptomic findings, reverse transcription quantitative polymerase chain reaction (RT-qPCR) was performed. Peripheral blood samples were collected from five healthy individuals and five patients with IS who were admitted to the Department of Neurosurgery at Qilu Hospital (Qingdao). The inclusion criteria were as follows: diagnosis of AIS confirmed by imaging; symptom onset within 72 h before admission; age ≥ 18 years; provision of informed consent. The exclusion criteria were as follows: presence of other severe neurological disorders, autoimmune diseases, or active infections; recent use of immunosuppressive or anticoagulant agents. The healthy controls had no history of cardiovascular or cerebrovascular diseases and were matched with the IS patients by age and sex. The blood samples were obtained within 24 h of admission, and the plasma was immediately separated and stored at −80° C until RNA extraction. The total RNA was extracted using TRIzol reagent (Ambion, Austin, TX, United States) according to manufacturer instructions. Reverse transcription was subsequently performed using a First Strand cDNA Synthesis Kit (Servicebio, Wuhan, China). RT-qPCR was conducted with 2× Universal Blue SYBR Green qPCR Master Mix (Servicebio, Wuhan, China) as per manufacturer protocols. The primer sequences used in this step are listed in Supplementary Table S1. The relative expression levels of the biomarkers were calculated using the 2^−ΔΔCq^ method with GAPDH as the internal control (Livak and Schmittgen, 2001).

### 2.12 Ethical approval

This study was conducted in accordance with the guidelines of the Declaration of Helsinki and was approved by the Medical Ethics Committee of Qilu Hospital, Shandong University (approval number: KYLL-KS-2025052; date: 23 January 2025). Written informed consent was obtained from all participants.

## 3 Results

### 3.1 Recognition of DEGs and key module genes in IS

**FIGURE 1:**
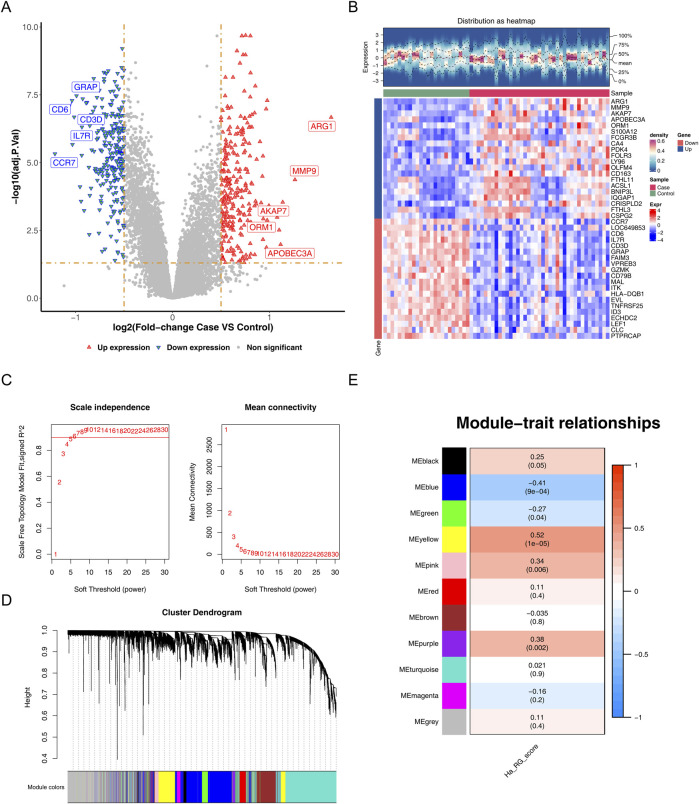
Identification of differentially expressed genes and key module genes in ischemic stroke (IS). **(A)** Volcano plot of differential gene expression, where red indicates significantly upregulated genes, blue indicates significantly downregulated genes, and gray indicates non-significant genes. **(B)** Heatmap of the differentially expressed genes. **(C)** Selection of the soft-thresholding power. **(D)** Dynamic tree-cutting for module detection. **(E)** Heatmap of the module–trait relationships; here, red indicates positive correlation and blue indicates negative correlation, with the color intensity reflecting the correlation strength.

A total of 518 DEGs were identified in the IS samples, including 272 upregulated and 246 downregulated genes (Figure 1A). The top-20 upregulated and top-20 downregulated DEGs are presented in Figure 1B. WGCNA was performed to identify modules that were closely associated with the ssGSEA enrichment scores of the HARGs; no outlier samples were detected here (Supplementary Figure S1). The optimal soft-thresholding power was determined to be 8, for which R^2^ approached 0.9 (red line) and the average network connectivity approached zero (Figure 1C). A total of 10 co-expression modules were subsequently identified (Figure 1D), among which the MEyellow module showed the strongest correlation with the ssGSEA enrichment scores of the HARGs (|cor| = 0.52, *p* = 1e-05) (Figure 1E). Accordingly, 137 key module genes that met the criteria |GS| ≥ 0.2 and |MM| ≥ 0.8 were selected for further analyses.

### 3.2 Identification and functional enrichment of the HAR-DEGs

**FIGURE 2:**
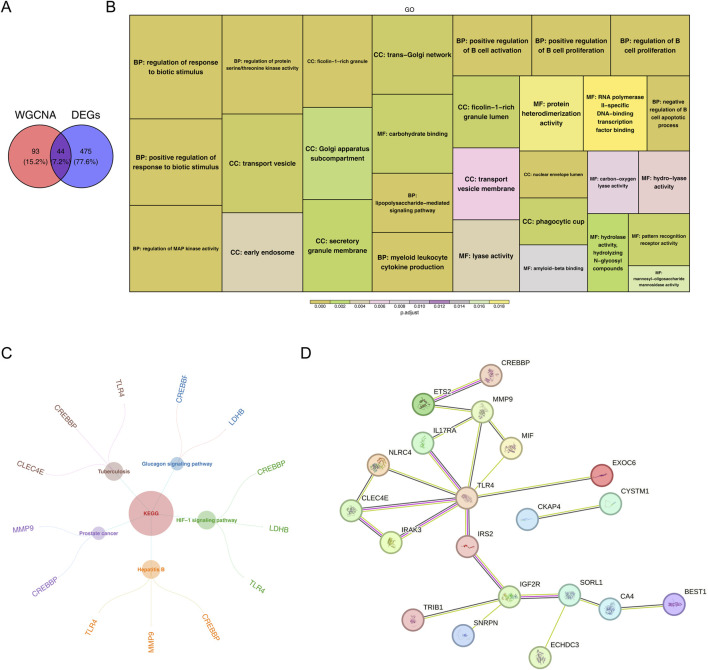
Identification and functional enrichment of the histone-acetylation-related differentially expressed genes (HAR-DEGs). **(A)** Venn diagram showing the HAR-DEGs. **(B)** Gene Ontology enrichment analysis of the HAR-DEGs. **(C)** Kyoto Encyclopedia of Genes and Genomes pathway enrichment analysis of the HAR-DEGs. **(D)** Protein–protein interaction network.

Intersection of the DEGs with the key module genes yielded 44 HAR-DEGs (Figure 2A). Next, functional enrichment analysis was conducted to explore the potential roles of these HAR-DEGs in IS. GO analysis indicated that the HAR-DEGs were primarily involved in the biological processes “regulation of MAP kinase activity” and “regulation of B-cell proliferation” (Figure 2B); KEGG analysis showed significant enrichments for the “HIF-1 signaling pathway” and “glucagon signaling pathway” (Figure 2C). In addition, the PPI network revealed extensive interactions among these genes, with *TLR4* serving as a central hub (Figure 2D).

### 3.3 Screening candidate biomarkers associated with histone acetylation regulatory networks in IS

**FIGURE 3:**
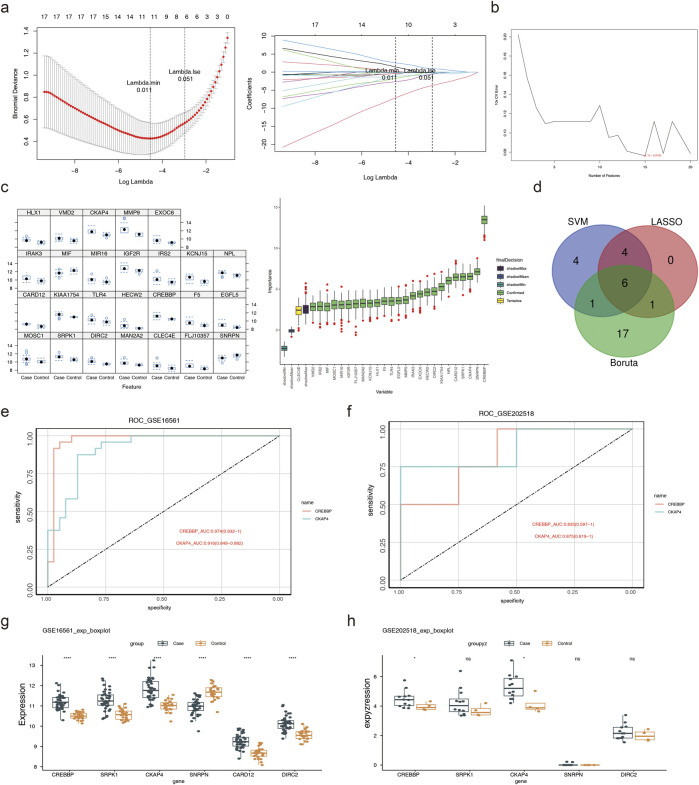
Biomarker screening. **(a)** LASSO regression analysis. **(b)** Relationship between support vector machine (SVM) generalization error and number of selected features. **(c)** Feature importance derived from the Boruta algorithm. **(d)** Venn diagram of the selected feature genes. **(e)** Receiver operating characteristics (ROC) curves of the candidate genes in the GSE16561 training set. **(f)** ROC curves of the candidate genes in the GSE202518 validation set. **(g)** Expression levels of the candidate genes in GSE16561, showing upregulation of *CREBBP* and *CKAP4* in the IS group compared to controls; ****p < 0.0001. **(h)** Expression levels of the candidate genes in GSE202518, confirming consistent upregulation of *CREBBP* and *CKAP4* in IS; ns, not significant; *p < 0.05.

To identify the key genes, LASSO analysis retained 11 feature genes with non-zero regression coefficients (Figure 3a). The SVM-RFE and Boruta methods further helped identify 15 and 25 feature genes, respectively (Figures 3b,c). Integration of the three algorithms yielded six candidate genes: *CREBBP*, *SRPK1*, *CKAP4*, *SNRPN*, *CARD12*, and *DIRC2* (Figure 3d). Among these, *CREBBP* and *CKAP4* demonstrated significant diagnostic performances with the validation dataset (AUC > 0.7), and their upregulated expression in IS was observed consistently for both the training and validation sets, supporting their identification as candidate biomarkers associated with the histone acetylation regulatory networks (Figures 3e–h). Notably, both *CREBBP* and *CKAP4* were significantly upregulated in the IS group compared to the normal group.

### 3.4 Immune microenvironment of the biomarkers

**FIGURE 4:**
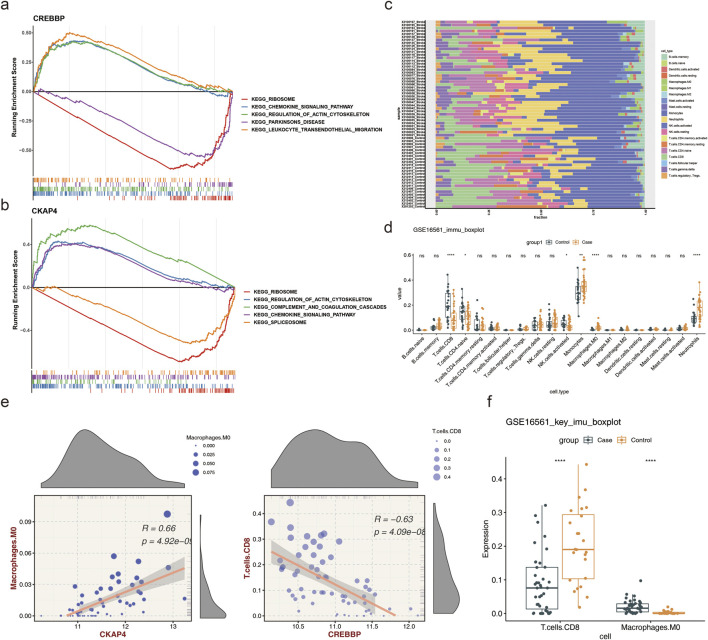
Gene set enrichment analysis (GSEA) and immune infiltration analysis. **(a)** GSEA enrichment plot for *CREBBP*. **(b)** GSEA enrichment plot for *CKAP4*. **(c)** Proportions of immune cell infiltration. **(d)** Differences in immune cell enrichment scores between the study groups; ns, not significant; *p < 0.05; **p < 0.01; ****p < 0.0001. **(e)** Correlations between the biomarkers and most relevant immune cells. **(f)** Differences in the enrichment scores of the most relevant immune cells; ****p < 0.0001.

GSEA was performed to explore the potential biological roles of *CREBBP* and *CKAP4* in IS. KEGG analysis showed that both biomarkers were significantly enriched for the immune-related pathways, including “ribosome” and “complement and coagulation cascades” (Figures 4a,b; Supplementary Table S2). The immune microenvironment in IS was subsequently characterized by estimating the infiltration levels of 22 types of immune cells (Figure 4c); here, six immune cell populations showed significant differences between the IS and normal groups (Figure 4d). Compared to the normal group, the IS group exhibited markedly increased proportions of monocytes, macrophages, and neutrophils along with significantly reduced numbers of CD8^+^ T cells, CD4^+^ T cells, and natural killer (NK) cells. Similar findings were obtained using the xCell algorithm (Supplementary Figure S2), which support the reliability of the CIBERSORT results. Correlation analysis revealed that *CREBBP* (cor = −0.63, *p* = 4.09e-08) and *CKAP4* (cor = −0.55, *p* = 3e-06) were negatively correlated with the CD8^+^ T cell scores, while *CREBBP* (cor = 0.57, *p* = 1e-06) and *CKAP4* (cor = 0.66, *p* = 4.92e-09) were positively correlated with the M0 macrophage scores (Figure 4e; Supplementary Figure S3). Notably, the infiltration levels of these two immune cell types differed significantly between the IS and normal groups (Figure 4f).

### 3.5 Analyses of regulatory network and potential therapeutic agents

**FIGURE 5:**
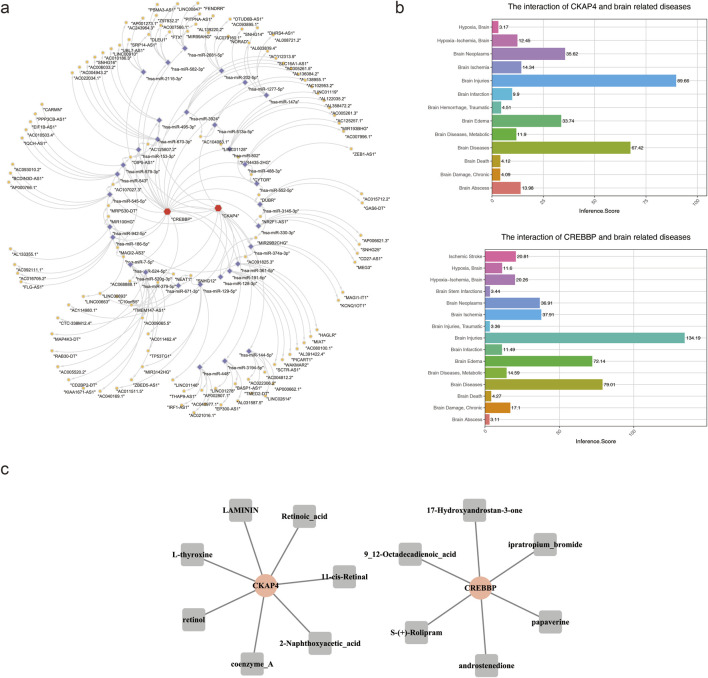
Competing endogenous RNA (ceRNA) network and drug prediction. **(a)** ceRNA network of the candidate biomarkers, where red indicates mRNAs, purple indicates miRNAs, and yellow indicates lncRNAs. **(b)** Disease association analysis. **(c)** Predicted therapeutic compound networks for IS based on *CREBBP* and *CKAP4*.

We constructed an lncRNA–miRNA–mRNA regulatory network to investigate the upstream regulatory mechanisms of *CREBBP* and *CKAP4*; the analysis suggested that AL133355.1 may regulate *CREBBP* expression via MIR100HG (Figure 5a), whereas SNHG25 may influence *CKAP4* expression through NR2F1-AS1. Disease association analysis further indicated that both biomarkers were closely related to brain injury and neurological disorders (Figure 5b). To identify the potential therapeutic targets, we predicted the drug–target interactions for *CREBBP* and *CKAP4* using DSigDB. *CREBBP* was significantly associated with six bioactive compounds, including retinoic acid, L-thyroxine, and S-(+)-rolipram, while *CKAP4* was predicted to interact with seven compounds, such as 2-naphthoxyacetic acid, 9,12-octadecadienoic acid, and androstenedione (Figure 5c). All predicted agents are pharmacologically active compounds, distinguishing them from non-specific environmental exposures.

### 3.6 Validating *CREBBP* and *CKAP4* expression in IS

**FIGURE 6:**
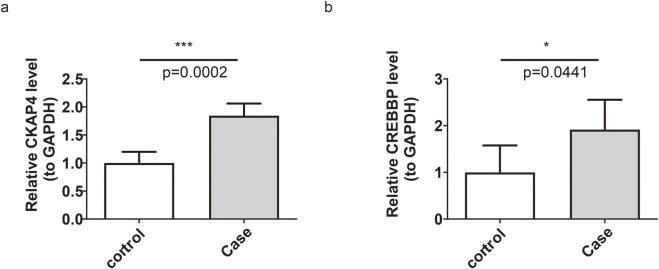
Reverse transcription quantitative polymerase chain reaction validation of the biomarker expression. **(a)** *CKAP4* expression level; ***p < 0.001. **(b)** *CREBBP* expression level; *p < 0.05.

The RT-qPCR results demonstrated that the expression levels of *CREBBP* and *CKAP4* were significantly higher in the IS group than the normal group, consistent with the transcriptomic findings and further supporting the reliability of the results (Figure 6).

## 4 Discussion

IS remains a major global health challenge; the neurological impairment and socioeconomic burden imposed by IS highlight the urgent need for more effective preventive and therapeutic strategies (Zhao et al., 2022). Through integrated bioinformatics analyses combined with clinical validation, we identified *CREBBP* and *CKAP4* as the HAR-DEGs in IS. Both genes were significantly upregulated in the peripheral blood samples from patients with IS and were jointly enriched in the “complement and coagulation cascades” pathway. In addition, their expression levels showed significant associations with the infiltration of CD8^+^ T cells and M0 macrophages. Based on these findings, we propose an integrative hypothesis that *CREBBP* and *CKAP4* may coordinately regulate post-stroke thromboinflammatory responses through epigenetic mechanisms, thereby forming an “epigenetic-immune-thrombotic” interaction axis.

The CREB-binding protein (CREBBP or CBP) is a core member of the KAT3 family of lysine acetyltransferases that plays a central role in chromatin remodeling and transcriptional regulation by acetylating both histone proteins (e.g., H3 and H4) and non-histone substrates (Dutto et al., 2018; White et al., 2024). Its functional relevance in IS extends far beyond general epigenetic modifications (Connelly et al., 2023; Liu R. et al., 2025). Although CREBBP reportedly exhibits antitumor activity (White et al., 2024), this aspect is secondary to its pathophysiological significance in IS. The most critical role of CREBBP in ischemic injury involves regulation of neuronal survival, synaptic plasticity, and ischemic tolerance, primarily through its function as a key downstream coactivator in the CREB signaling pathway, which is essential for post-ischemic brain recovery. Upon binding to phosphorylated CREB, CREBBP acetylates H3/H4 at the promoter regions of neuroprotective genes such as brain-derived neurotrophic factor (*BDNF*) and B-cell lymphoma/leukemia-2 (*BCL-2*), thereby enhancing their transcription (Yildirim et al., 2014; Narasimhamurthy et al., 2022). Preclinical evidence further indicates that ischemic preconditioning enhances CREB pathway activity by increasing *CREBBP* expression, leading to reduced neuronal apoptosis and improved neurological recovery (Yildirim et al., 2014). Beyond CREB-mediated neuroprotection, *CREBBP* contributes to the pathophysiology of IS via additional mechanisms, including DNA damage repair and neurovascular remodeling. In the context of genomic maintenance, *CREBBP* acetylates key components of the base excision repair and non-homologous end joining pathways, thereby preserving neuronal genomic stability under ischemic stress (Dutto et al., 2018; Ramadan et al., 2025). In terms of vascular repair, the aPKC–CREBBP signaling axis has been shown to directly regulate post-stroke angiogenesis and functional recovery, whereas inhibition of CREBBP acetyltransferase activity exacerbates ischemic brain injury (Gouveia et al., 2017). *CREBBP* exerts substantial influences on both the pathological progression and clinical outcomes of IS through the integration of multiple protective mechanisms, including CREB pathway activation, DNA repair, and neurovascular remodeling.

Cytoskeleton-associated protein 4 (CKAP4) is a type II transmembrane protein that is predominantly localized in rough endoplasmic reticulum, where it participates in intracellular protein trafficking and cytoskeletal organization (Schweizer et al., 1995). There is currently no direct experimental evidence supporting the role of *CKAP4* in histone acetylation regulation. In the present study, *CKAP4* was categorized as a gene associated with histone acetylation regulation based on its strong co-expression with established histone acetylation regulators in IS transcriptomic datasets. This co-expression pattern suggests a potential functional link between *CKAP4* and the histone acetylation regulatory network in the pathological context of stroke, although the underlying molecular mechanisms remain unclear. Emerging evidence indicates that *CKAP4* is involved in neurological disorders. Cytoskeletal reorganization is a key pathological feature in neurodegenerative diseases, such as Alzheimer’s disease and Parkinson’s disease, and *CKAP4* has been implicated in these processes (Shah and Rossie, 2018). In addition, *CKAP4*-mediated alterations in the platelet cytoskeletal architecture may affect thrombus permeability, potentially influencing the efficacy of thrombolytic therapy in IS (Schartz et al., 2023). These findings suggest that *CKAP4* may contribute to stroke pathogenesis primarily through non-epigenetic mechanisms, particularly those related to cytoskeletal dynamics and intracellular transport. Accordingly, *CKAP4* should be regarded as a candidate gene showing transcriptional co-regulation with histone-acetylation-related genes in stroke rather than a confirmed direct epigenetic regulator. Further mechanistic studies are required to determine whether *CKAP4* affects histone acetylation indirectly, for example, through the cytoskeleton-dependent regulation of nuclear transport or protein compartmentalization, or whether its roles in ischemic injury and repair are independent of epigenetic modulations.

GSEA further indicated that elevated expression of *CREBBP* and *CKAP4* were associated with enrichment of the “complement and coagulation cascades” pathway. For *CREBBP* as a well-characterized HAT, this association may reflect the direct transcriptional co-activation of pathway-related genes. Previous studies have demonstrated that *CREBBP* regulates the expression of complement components such as C3 and C5 through histone acetylation at the promoter regions (Cui et al., 2025). In contrast, the epigenetic role of *CKAP4* remains uncertain, and its association with this pathway is likely indirect. *CKAP4* may influence the subcellular localization and functional activities of HDACs through its roles in protein trafficking or cytoskeletal remodeling (Tan et al., 2021; Srivastava et al., 2022), thereby indirectly affecting the acetylation levels at the promoter regions of complement-related genes (Ma et al., 2019). Thus, although both genes are linked to the histone acetylation regulatory network, *CREBBP* is a well-defined epigenetic regulator, whereas *CKAP4* should be considered a correlated candidate gene with an unresolved mechanism that warrants further investigation.

The involvement of the immune system has been increasingly recognized in the pathogenesis of IS. In the present study, monocytes, macrophages, and neutrophils were significantly increased in the IS group, whereas CD4^+^ T cells, CD8^+^ T cells, and NK cells were markedly reduced compared to the normal control group. Previous studies have shown that CD8^+^ T cells aggravate post-ischemic neuroinflammation through the secretion of interferon-γ (IFN-γ), which subsequently enhances tumor necrosis factor-α (TNF-α) production (Gelderblom et al., 2012). Within the macrophage population, the M1 phenotype exhibits proinflammatory properties, whereas the M2 phenotype exerts anti-inflammatory and neuroprotective effects (Guruswamy and ElAli, 2017). The dynamic balance between these phenotypes at different post-stroke stages critically influences the progression and severity of brain injury. Notably, *CREBBP* expression is negatively correlated with CD8^+^ T cell infiltration, while *CKAP4* expression is positively associated with M0 macrophage infiltration, suggesting potential roles for these molecules in modulating the immune microenvironment in IS. However, the molecular mechanisms underlying these associations require further experimental validation. Overall, these findings reveal novel relationships between *CREBBP* and *CKAP4* expression and the infiltration of specific immune cell subsets, providing mechanistic insights on their immunoregulatory functions for future studies.

The lncRNA–miRNA–mRNA regulatory network showed *CREBBP* as a downstream target of MIR100HG and *CKAP4* as a downstream target of NR2F1-AS1. MIR100HG functions as a key regulatory lncRNA within the TGFβ signaling pathway. In IS, MIR100HG promotes TGFβ1 secretion by forming a ternary ribonucleoprotein complex with the TGFβ1 mRNA and RNA-binding protein HuR, thereby modulating the dual stage-dependent effects of TGFβ signaling characterized by early neuroprotection and subsequent glial scar formation (Papoutsoglou et al., 2021). In addition, MIR100HG acts as a molecular integrator across multiple signaling pathways, including Wnt and Hippo, to collectively influence ischemic outcomes (Ghafouri-Fard et al., 2023). NR2F1-AS1 is known to activate the AKT–mTOR signaling axis through transcriptional upregulation of its sense gene NR2F1 (Liu et al., 2022b). Given the established roles of AKT–mTOR signaling in regulating neuronal survival, autophagy, angiogenesis, and neuroinflammation after IS (Zhu et al., 2021; Liu et al., 2025c), NR2F1-AS1 may play a critical role in post-ischemic neural repair. In summary, MIR100HG and NR2F1-AS1 exert regulatory effects on key pathophysiological pathways in IS and are promising targets for therapeutic intervention.

DSigDB analysis was performed to predict potential bioactive compounds targeting these two genes. Among the *CREBBP*-associated compounds, including pyrethroids and cannabinoids, certain agents may aggravate neurotoxicity and increase stroke risk by enhancing oxidative stress, whereas retinoic acid and S-(+)-rolipram exhibit neuroprotective or anti-inflammatory effects (Kang and Koh, 2023; Jiang et al., 2024). Among the *CKAP4*-related agents, tobacco smoke pollutants, acetaminophen, and progesterone have been reported to modulate inflammation and oxidative stress by regulating macrophage function (Jones et al., 2008; Barnes, 2016; Du et al., 2016); in addition, linoleic acid demonstrates anti-inflammatory potential mediated by macrophage modulation (Qin et al., 2024). These *in silico* predictions provide mechanistic insights into the roles of *CREBBP* and *CKAP4* in the pathogenesis of IS and highlight candidate therapeutic interventions. Overall, epigenetic upregulation of *CREBBP* and *CKAP4*, either directly or indirectly, can enhance activation of the complement and coagulation cascades synergistically while differentially regulating CD8^+^ T cell and macrophage functions. This coordinated dysregulation sustains a self-perpetuating cycle of thromboinflammation, ultimately leading to neuronal injury. Future investigations should experimentally evaluate the functional effects of *CREBBP* and *CKAP4* on cell viability, apoptosis, histone acetylation, and downstream gene expression in relevant neural cell types, including neurons, brain microvascular endothelial cells, and microglia, using integrated strategies that combine genetic manipulation (e.g., knockdown or overexpression) with oxygen–glucose deprivation (OGD) models. Such studies will clarify the direct regulatory roles of these genes in the pathophysiology of IS and establish a solid experimental basis for targeted therapeutic development.

Lastly, we note that this study has several limitations. The training set (GSE16561: 39 controls vs. 24 IS cases) and validation set (GSE202518: 4 controls vs. 12 IS cases) were relatively small, and RT-qPCR validation was conducted in only five paired clinical samples, which could potentially reduce the statistical power and limit generalizability. In addition, the associations of *CREBBP* and *CKAP4* with IS, their correlations with immune infiltration, and their positions within the predicted regulatory networks were derived exclusively from transcriptomic analyses and have not yet been causally validated in cellular or animal models. The identification of candidate therapeutic compounds was also based solely on database-derived correlations, and their regulatory mechanisms and therapeutic efficacies need to be experimentally confirmed. Notably, although *CREBBP* is a well-characterized HAT, its enzymatic activity has not been directly assessed in blood samples from patients with IS, nor are there any characterizations of its *in vivo* histone acetylation targets. Similarly, the proposed involvement of *CKAP4* in the histone acetylation network is supported primarily by the co-expression analysis, with no direct experimental evidence for an epigenetic regulatory role; therefore, its functional significance remains unclear. Future works will address these limitations through multicenter collaborations to expand the cohort size and enable rigorous validations in larger independent populations. Gain- and loss-of-function studies will also be performed in relevant cell systems and animal models of IS. Comprehensive characterization of the epigenetic regulatory landscape, including *CREBBP*-mediated histone modifications and downstream signaling pathways, will be conducted using chromatin immunoprecipitation (ChIP), dual-luciferase reporter assays, and complementary techniques. Concurrently, the pharmacological effects of the predicted therapeutic compounds will be systematically evaluated *in vitro* and *in vivo* to facilitate translation into clinically relevant treatment strategies.

## 5 Conclusion

Integrated bioinformatics analyses were performed to identify two candidate biomarkers *CREBBP* and *CKAP4* that are co-expressed within a histone-acetylation-related gene network in IS. Both genes were found to be associated with immune-related pathways, including complement and coagulation cascades, and were correlated with the infiltration of CD8^+^ T cells and M0 macrophages. These results suggest that *CREBBP* and *CKAP4* are closely linked to the infiltration of key immune cell populations in the IS immune microenvironment and may serve as potential immune-related biomarkers to provide a basis for further mechanistic investigations.

## Data Availability

The datasets (GSE16561 and GSE202518) analyzed in this study are publicly available in the Gene Expression Omnibus (GEO) repository (https://www.ncbi.nlm.nih.gov/gds). Additional data were obtained from the miRDB (http://mirdb.org), TargetScan (http://www.targetscan.org), and CTD (http://ctdbase.org) databases.

## Glossary

AIS: acute ischemic stroke
AKT: protein kinase B
aPKC/APKC: atypical protein kinase C
ATM: ataxia–telangiectasia mutated
AUC: area under the curve
BCL-2: B-cell lymphoma 2
BDNF: brain-derived neurotrophic factor
BH: Benjamini–Hochberg
CBP/CREBBP: CREB-binding protein
cDNA: complementary DNA
ceRNA: competing endogenous RNA
cGAS: cyclic GMP–AMP synthase
CKAP4: cytoskeleton-associated protein 4
CREB: cAMP response-element-binding protein
DEG: differentially expressed gene
DSigDB: Drug Signatures Database
FC: fold change
FDR: false discovery rate
GAPDH: glyceraldehyde-3-phosphate dehydrogenase
GEO: Gene Expression Omnibus
GO: Gene Ontology
GPL: GEO platform (platform accession)
GSEA: gene set enrichment analysis
H3: histone H3
H4: histone H4
HAR-DEGs: histone-acetylation-related differentially expressed genes
HARGs: histone acetylation regulatory genes
HAT: histone acetyltransferase
HDAC: histone deacetylase
HDAC3: histone deacetylase 3
HIF-1: hypoxia-inducible factor 1
I/R: ischemia–reperfusion
IFN-γ: interferon gamma
IL: interleukin
IS: ischemic stroke
JNK: c-Jun N-terminal kinase
KEGG: Kyoto Encyclopedia of Genes and Genomes
LASSO: least-absolute shrinkage and selection operator
lncRNA: long non-coding RNA
MAP: mitogen-activated protein
miRNA: microRNA
mRNA: messenger RNA
mTOR: mechanistic target of rapamycin
NK: natural killer
OGD: oxygen–glucose deprivation
PAR2: protease-activated receptor 2
PCR: polymerase chain reaction
PI3K: phosphoinositide 3-kinase
PPI: protein–protein interaction
RNA-seq: RNA sequencing
ROC: receiver operating characteristic
RT-qPCR: reverse transcription quantitative polymerase chain reaction
ssGSEA: single-sample gene set enrichment analysis
STING: stimulator of interferon genes
STRING: Search Tool for the Retrieval of Interacting Genes/Proteins
SVM-RFE: support vector machine recursive feature elimination
SYBR: SYBR Green fluorescent dye
TGFβ: transforming growth factor beta
TLR4: Toll-like receptor 4
TNF-α: tumor necrosis factor alpha
TRIzol: TRIzol reagent
WGCNA: weighted gene co-expression network analysis
xCell: xCell cell-type enrichment analysis method

## References

[B1] Aran D. (2020). Cell-type enrichment analysis of bulk transcriptomes using xCell. Methods Mol. Biol. 2120, 263–276. 10.1007/978-1-0716-0327-7_19 32124326

[B2] Barnes P. J. (2016). Inflammatory mechanisms in patients with chronic obstructive pulmonary disease. J. Allergy Clin. Immunol. 138 (1), 16–27. 10.1016/j.jaci.2016.05.011 27373322

[B3] Chen X. M. Zhao Y. Wu X. D. Wang M. J. Yu H. Lu J. J. (2019). Novel findings from determination of common expressed plasma exosomal microRNAs in patients with psoriatic arthritis, psoriasis vulgaris, rheumatoid arthritis, and gouty arthritis. Discov. Med. 28 (151), 47–68. 31465725

[B4] Connelly J. A. Zhang X. Chen Y. Chao Y. Shi Y. Jacob T. C. (2023). Protein kinase D2 confers neuroprotection by promoting AKT and CREB activation in ischemic stroke. Neurobiol. Dis. 187, 106305. 10.1016/j.nbd.2023.106305 37730136 PMC10836334

[B5] Cui G. Ley M. Mechaly A. E. Bui L. C. Michail C. Berthelet J. (2025). Structural and functional characterization of CREB-binding protein (CREBBP) as a histone propionyltransferase. J. Biol. Chem. 301 (8), 110444. 10.1016/j.jbc.2025.110444 40615044 PMC12305233

[B6] Du K. Ramachandran A. Jaeschke H. (2016). Oxidative stress during acetaminophen hepatotoxicity: sources, pathophysiological role and therapeutic potential. Redox Biol. 10, 148–156. 10.1016/j.redox.2016.10.001 27744120 PMC5065645

[B7] Dutto I. Scalera C. Prosperi E. (2018). CREBBP and p300 lysine acetyl transferases in the DNA damage response. Cell Mol. Life Sci. 75 (8), 1325–1338. 10.1007/s00018-017-2717-4 29170789 PMC11105205

[B8] Fang Y. Zhao J. Guo X. Dai Y. Zhang H. Yin F. (2022). Establishment, immunological analysis, and drug prediction of a prognostic signature of ovarian cancer related to histone acetylation. Front. Pharmacol. 13, 947252. 10.3389/fphar.2022.947252 36172179 PMC9510621

[B9] Feigin V. L. Stark B. A. Johnson C. O. Roth G. A. Bisignano C. Abady G. G. (2021). Global, regional, and national burden of stroke and its risk factors, 1990–2019: a systematic analysis for the global Burden of Disease Study 2019. Lancet Neurol. 20 (10), 795–820. 10.1016/s1474-4422(21)00252-0 34487721 PMC8443449

[B10] Gelderblom M. Weymar A. Bernreuther C. Velden J. Arunachalam P. Steinbach K. (2012). Neutralization of the IL-17 axis diminishes neutrophil invasion and protects from ischemic stroke. Blood 120 (18), 3793–3802. 10.1182/blood-2012-02-412726 22976954

[B11] Ghafouri-Fard S. Harsij A. Farahzadi H. Hussen B. M. Taheri M. Mokhtari M. (2023). A concise review on the role of MIR100HG in human disorders. J. Cell. Mol. Med. 27 (16), 2278–2289. 10.1111/jcmm.17875 37487022 PMC10424294

[B12] Gong H. Liu J. Chen N. Zhao H. He B. Zhang H. (2025). EDN1 and NTF3 in keloid pathogenesis: computational and experimental evidence as novel diagnostic biomarkers for fibrosis and inflammation. Front. Genet. 16, 1516451. 10.3389/fgene.2025.1516451 40051702 PMC11882859

[B13] Gouveia A. Seegobin M. Kannangara T. S. He L. Wondisford F. Comin C. H. (2017). The aPKC-CBP pathway regulates post-stroke neurovascular remodeling and functional recovery. Stem Cell Rep. 9 (6), 1735–1744. 10.1016/j.stemcr.2017.10.021 29173896 PMC5785704

[B14] Gu Z. Eils R. Schlesner M. (2016). Complex heatmaps reveal patterns and correlations in multidimensional genomic data. Bioinformatics 32 (18), 2847–2849. 10.1093/bioinformatics/btw313 27207943

[B15] Guruswamy R. ElAli A. (2017). Complex roles of microglial cells in ischemic stroke pathobiology: new insights and future directions. Int. J. Mol. Sci. 18 (3), 496. 10.3390/ijms18030496 28245599 PMC5372512

[B16] Hao L. Xu Q. Yang G. Di M. (2025). Identifying and validating hypoxia- and metabolism-related hub genes and cell communication in atherosclerosis. Front. Cardiovasc. Med. 12, 1680482. 10.3389/fcvm.2025.1680482 41458991 PMC12740931

[B17] Herpich F. Rincon F. (2020). Management of Acute Ischemic stroke. Crit. Care Med. 48 (11), 1654–1663. 10.1097/ccm.0000000000004597 32947473 PMC7540624

[B18] Jiang S. Liu Z. Zhang X. Zhang R. Sun B. Gao C. (2024). Bioinformatics screening and verification of ischemic stroke-related key genes and drug prediction. Gen. Physiol. Biophys. 43 (5), 385–397. 10.4149/gpb_2024023 39140683

[B19] Jones L. A. Anthony J. P. Henriquez F. L. Lyons R. E. Nickdel M. B. Carter K. C. (2008). Toll-like receptor-4-mediated macrophage activation is differentially regulated by progesterone *via* the glucocorticoid and progesterone receptors. Immunology 125 (1), 59–69. 10.1111/j.1365-2567.2008.02820.x 18373668 PMC2526260

[B20] Kang J. B. Koh P. O. (2023). Retinoic acid has neuroprotective effects by modulating thioredoxin in ischemic brain damage and glutamate-exposed neurons. Neuroscience 521, 166–181. 10.1016/j.neuroscience.2023.04.028 37149281

[B21] Kang J. Choi Y. J. Kim I. K. Lee H. S. Kim H. Baik S. H. (2021). LASSO-Based machine learning Algorithm for prediction of lymph node metastasis in T1 colorectal cancer. Cancer Res. Treat. 53 (3), 773–783. 10.4143/crt.2020.974 33421980 PMC8291173

[B22] Li M. Wang J. Liu D. Huang H. (2018). High-throughput sequencing reveals differentially expressed lncRNAs and circRNAs, and their associated functional network, in human hypertrophic scars. Mol. Med. Rep. 18 (6), 5669–5682. 10.3892/mmr.2018.9557 30320389 PMC6236202

[B23] Liao Y. Cheng J. Kong X. Li S. Li X. Zhang M. (2020). HDAC3 inhibition ameliorates ischemia/reperfusion-induced brain injury by regulating the microglial cGAS-STING pathway. Theranostics 10 (21), 9644–9662. 10.7150/thno.47651 32863951 PMC7449914

[B24] Liu P. Xu H. Shi Y. Deng L. Chen X. (2020). Potential molecular mechanisms of plantain in the treatment of gout and hyperuricemia based on network pharmacology. Evid. Based Complement. Altern. Med. 2020, 3023127. 10.1155/2020/3023127 33149752 PMC7603577

[B25] Liu K. Geng Y. Wang L. Xu H. Zou M. Li Y. (2022a). Systematic exploration of the underlying mechanism of gemcitabine resistance in pancreatic adenocarcinoma. Mol. Oncol. 16 (16), 3034–3051. 10.1002/1878-0261.13279 35810469 PMC9394232

[B26] Liu Y. Chen S. Cai K. Zheng D. Zhu C. Li L. (2022b). Hypoxia-induced long noncoding RNA NR2F1-AS1 maintains pancreatic cancer proliferation, migration, and invasion by activating the NR2F1/AKT/mTOR axis. Cell Death Dis. 13 (3), 232. 10.1038/s41419-022-04669-0 35283481 PMC8918554

[B27] Liu L. Li X. Yang H. Xu F. Dong X. (2025a). Bioinformatic analysis of apoptosis-related genes in Preeclampsia using public transcriptomic and single-cell RNA sequencing datasets. J. Inflamm. Res. 18, 4785–4812. 10.2147/jir.S507660 40224388 PMC11992479

[B28] Liu R. Li J. Nian Q. Tu G. Wang Z. Zhang R. (2025b). CREB binding protein (CREBBP): Structure-based perspectives for the development of clinical inhibitors. Transl. Oncol. 61, 102507. 10.1016/j.tranon.2025.102507 40829266 PMC12391507

[B29] Liu T. Li X. Zhou X. Chen W. Wen A. Liu M. (2025c). PI3K/AKT signaling and neuroprotection in ischemic stroke: molecular mechanisms and therapeutic perspectives. Neural Regen. Res. 20 (10), 2758–2775. 10.4103/nrr.Nrr-d-24-00568 39435629 PMC11826468

[B30] Livak K. J. Schmittgen T. D. (2001). Analysis of relative gene expression data using real-time quantitative PCR and the 2(-Delta Delta C(T)) Method. Methods 25 (4), 402–408. 10.1006/meth.2001.1262 11846609

[B31] Ma Y. Liu Y. Zhang Z. Yang G. Y. (2019). Significance of complement System in ischemic stroke: a comprehensive review. Aging Dis. 10 (2), 429–462. 10.14336/ad.2019.0119 31011487 PMC6457046

[B32] Narasimhamurthy R. K. Andrade D. Mumbrekar K. D. (2022). Modulation of CREB and its associated upstream signaling pathways in pesticide-induced neurotoxicity. Mol. Cell Biochem. 477 (11), 2581–2593. 10.1007/s11010-022-04472-7 35596844 PMC9618525

[B33] Papoutsoglou P. Rodrigues-Junior D. M. Morén A. Bergman A. Pontén F. Coulouarn C. (2021). The noncoding *MIR100HG* RNA enhances the autocrine function of transforming growth factor β signaling. Oncogene 40 (21), 3748–3765. 10.1038/s41388-021-01803-8 33941855 PMC8154591

[B34] Qin Y. Li K. Zhang Q. Liu J. Xie Y. Zhang T. (2024). Linoleic acid inhibits lipopolysaccharide-induced inflammation by promoting TLR4 regulated autophagy in murine RAW264.7 macrophages. J. Appl. Biomed. 22 (4), 185–196. 10.32725/jab.2024.023 40033806

[B35] Rabinstein A. A. (2020). Update on treatment of acute Ischemic stroke. Contin. (Minneap Minn) 26 (2), 268–286. 10.1212/con.0000000000000840 32224752

[B36] Ramadan W. S. Ahmed S. B. M. Talaat I. M. Lozon L. Mouffak S. Gemoll T. (2025). The histone acetyltransferase CBP participates in regulating the DNA damage response through ATM after double-strand breaks. Genome Biol. 26 (1), 89. 10.1186/s13059-025-03528-3 40200339 PMC11980100

[B37] Rey F. Messa L. Pandini C. Maghraby E. Barzaghini B. Garofalo M. (2021). RNA-seq characterization of sex-differences in adipose tissue of obesity affected patients: computational analysis of differentially expressed coding and non-coding RNAs. J. Pers. Med. 11 (5), 352. 10.3390/jpm11050352 33924951 PMC8145808

[B38] Ritchie M. E. Phipson B. Wu D. Hu Y. Law C. W. Shi W. (2015). Limma powers differential expression analyses for RNA-sequencing and microarray studies. Nucleic Acids Res. 43 (7), e47. 10.1093/nar/gkv007 25605792 PMC4402510

[B39] Robin X. Turck N. Hainard A. Tiberti N. Lisacek F. Sanchez J. C. (2011). pROC: an open-source package for R and S+ to analyze and compare ROC curves. BMC Bioinforma. 12, 77. 10.1186/1471-2105-12-77 21414208 PMC3068975

[B40] Schartz D. Akkipeddi S. M. K. Rahmani R. Ellens N. Houk C. Kohli G. S. (2023). Ischemic stroke thrombus perviousness is associated with distinguishable proteomic features and susceptibility to ADAMTS13-Augmented thrombolysis. AJNR Am. J. Neuroradiol. 45 (1), 22–29. 10.3174/ajnr.A8069 38123915 PMC10756583

[B41] Schweizer A. Rohrer J. Slot J. W. Geuze H. J. Kornfeld S. (1995). Reassessment of the subcellular localization of p63. J. Cell Sci. 108 (Pt 6), 2477–2485. 10.1242/jcs.108.6.2477 7673362

[B42] Schweizer S. Meisel A. Märschenz S. (2013). Epigenetic mechanisms in cerebral ischemia. J. Cereb. Blood Flow. Metab. 33 (9), 1335–1346. 10.1038/jcbfm.2013.93 23756691 PMC3764391

[B43] Shah K. Rossie S. (2018). Tale of the good and the bad Cdk5: remodeling of the actin Cytoskeleton in the brain. Mol. Neurobiol. 55 (4), 3426–3438. 10.1007/s12035-017-0525-3 28502042 PMC6370010

[B44] Srivastava A. Banerjee J. Dubey V. Tripathi M. Chandra P. S. Sharma M. C. (2022). Role of altered expression, activity and sub-cellular distribution of various histone deacetylases (HDACs) in mesial temporal lobe Epilepsy with hippocampal sclerosis. Cell Mol. Neurobiol. 42 (4), 1049–1064. 10.1007/s10571-020-00994-0 33258018 PMC11441253

[B45] Tan H. Chen Z. Chen F. Xu W. Liu X. (2021). CKAP4 participates in tryptase-induced phenotypic conversion in atrial fibroblasts through PAR2/p38/JNK pathway. Am. J. Transl. Res. 13 (4), 2270–2282. 34017388 PMC8129387

[B46] Tu W. J. Zhao Z. Yin P. Cao L. Zeng J. Chen H. (2023). Estimated Burden of Stroke in China in 2020. JAMA Netw. Open 6 (3), e231455. 10.1001/jamanetworkopen.2023.1455 36862407 PMC9982699

[B47] Uzdensky A. B. Demyanenko S. (2021). Histone acetylation and deacetylation in ischemic stroke. Neural Regen. Res. 16 (8), 1529–1530. 10.4103/1673-5374.303024 33433467 PMC8323678

[B48] Wang Z. Zang C. Cui K. Schones D. E. Barski A. Peng W. (2009). Genome-wide mapping of HATs and HDACs reveals distinct functions in active and inactive genes. Cell 138 (5), 1019–1031. 10.1016/j.cell.2009.06.049 19698979 PMC2750862

[B49] White J. Derheimer F. A. Jensen-Pergakes K. O'Connell S. Sharma S. Spiegel N. (2024). Histone lysine acetyltransferase inhibitors: an emerging class of drugs for cancer therapy. Trends Pharmacol. Sci. 45 (3), 243–254. 10.1016/j.tips.2024.01.010 38383216

[B50] Wu T. Hu E. Xu S. Chen M. Guo P. Dai Z. (2021). clusterProfiler 4.0: a universal enrichment tool for interpreting omics data. Innov. (Camb) 2 (3), 100141. 10.1016/j.xinn.2021.100141 34557778 PMC8454663

[B51] Wu X. Qin K. Iroegbu C. D. Xiang K. Peng J. Guo J. (2022). Genetic analysis of potential biomarkers and therapeutic targets in ferroptosis from coronary artery disease. J. Cell Mol. Med. 26 (8), 2177–2190. 10.1111/jcmm.17239 35152560 PMC8995456

[B52] Yildirim F. Ji S. Kronenberg G. Barco A. Olivares R. Benito E. (2014). Histone acetylation and CREB binding protein are required for neuronal resistance against ischemic injury. PLoS One 9 (4), e95465. 10.1371/journal.pone.0095465 24748101 PMC3991684

[B53] Yu G. Wang L. G. Han Y. He Q. Y. (2012). clusterProfiler: an R package for comparing biological themes among gene clusters. Omics 16 (5), 284–287. 10.1089/omi.2011.0118 22455463 PMC3339379

[B54] Yu X. Xu H. Xing Y. Sun D. Li D. Shi J. (2025). Identifying essential hub genes and circRNA-Regulated ceRNA networks in hepatocellular carcinoma. Int. J. Mol. Sci. 26 (4), 1408. 10.3390/ijms26041408 40003874 PMC11855757

[B55] Yuan H. Lv Y. Fan P. Jia P. Wang K. Hu K. (2025). Identifying biomarkers of hepatic fatty acid metabolism disorder in sevoflurane-induced brain developmental injury by bioinformatics analysis. Front. Mol. Neurosci. 18, 1369365. 10.3389/fnmol.2025.1369365 40980340 PMC12443771

[B56] Yue S. Li S. Huang X. Liu J. Hou X. Zhao Y. (2022). Machine learning for the prediction of acute kidney injury in patients with sepsis. J. Transl. Med. 20 (1), 215. 10.1186/s12967-022-03364-0 35562803 PMC9101823

[B57] Zhang H. Li S. Deng Z. Wang Y. (2024). Molecular differences in glomerular compartment to distinguish Immunoglobulin A nephropathy and lupus nephritis. J. Inflamm. Res. 17, 11357–11373. 10.2147/jir.S496138 39722731 PMC11669337

[B58] Zhao S. Guo Y. Sheng Q. Shyr Y. (2014). Advanced heat map and clustering analysis using heatmap3. Biomed. Res. Int. 2014, 986048. 10.1155/2014/986048 25143956 PMC4124803

[B59] Zhao Y. Zhang X. Chen X. Wei Y. (2022). Neuronal injuries in cerebral infarction and ischemic stroke: from mechanisms to treatment. Int. J. Mol. Med. 49 (2), 15. 10.3892/ijmm.2021.5070 34878154 PMC8711586

[B60] Zheng Z. Zhang Q. Wu W. Xue Y. Liu S. Chen Q. (2021). Identification and validation of a ferroptosis-related long non-coding RNA signature for predicting the outcome of lung adenocarcinoma. Front. Genet. 12, 690509. 10.3389/fgene.2021.690509 34367250 PMC8339970

[B61] Zhu H. Zhang Y. Zhong Y. Ye Y. Hu X. Gu L. (2021). Inflammation-Mediated angiogenesis in ischemic stroke. Front. Cell. Neurosci. 15, 652647. 10.3389/fncel.2021.652647 33967696 PMC8096981

[B62] Zuo Z. G. Zhang X. F. Ye X. Z. Zhou Z. H. Wu X. B. Ni S. C. (2016). Bioinformatic analysis of RNA-seq data unveiled critical genes in rectal adenocarcinoma. Eur. Rev. Med. Pharmacol. Sci. 20 (14), 3017–3025. 27460729

