# Supplementary figures and images for: Exploring histone acetylation in ischemic stroke: CREBBP and CKAP4 as candidate biomarkers linked to histone acetylation networks
Source: Front Pharmacol. 2026 Apr 2;17:1727813. doi: 10.3389/fphar.2026.1727813 (PMC13083126; doi:10.3389/fphar.2026.1727813)

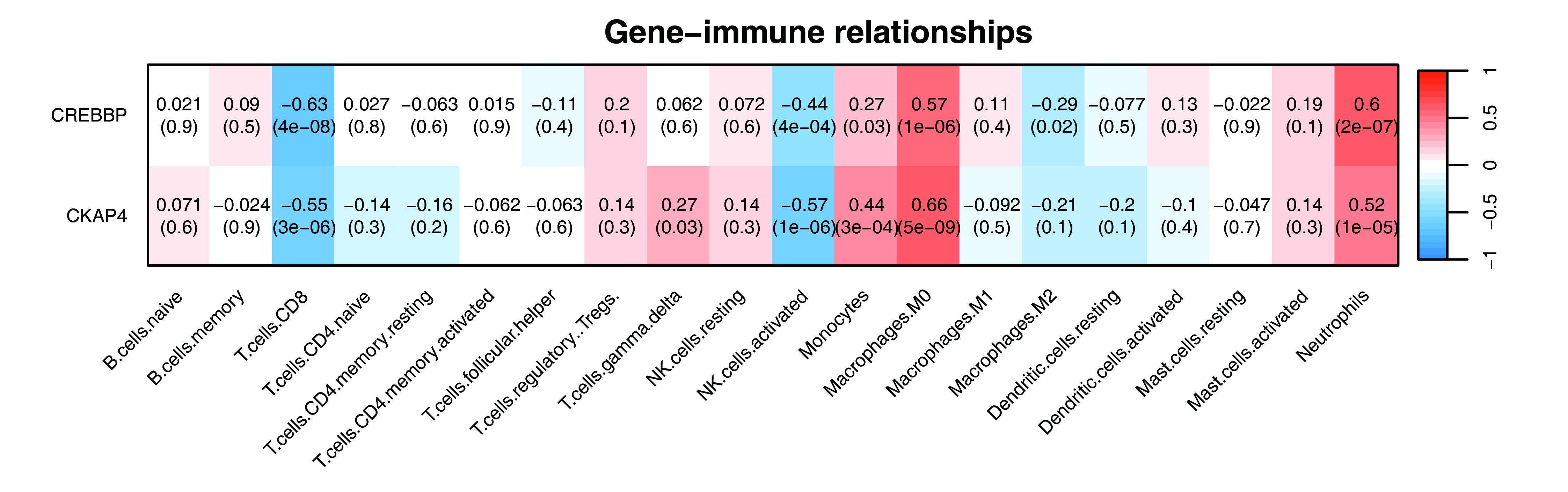

Supplement: Supplementary file 2 [file Image3.tif]

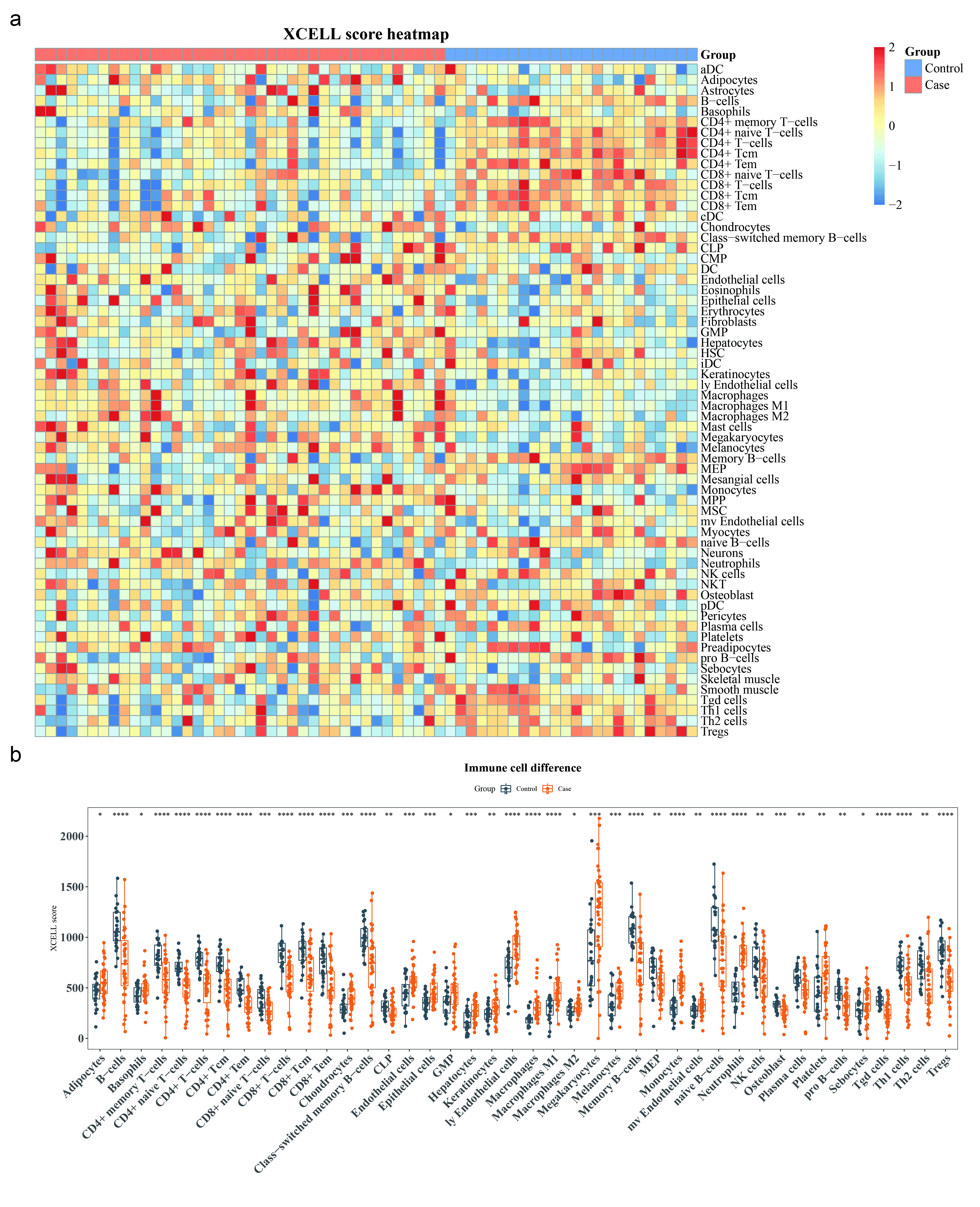

Supplement: Supplementary file 3 [file Image2.tif]

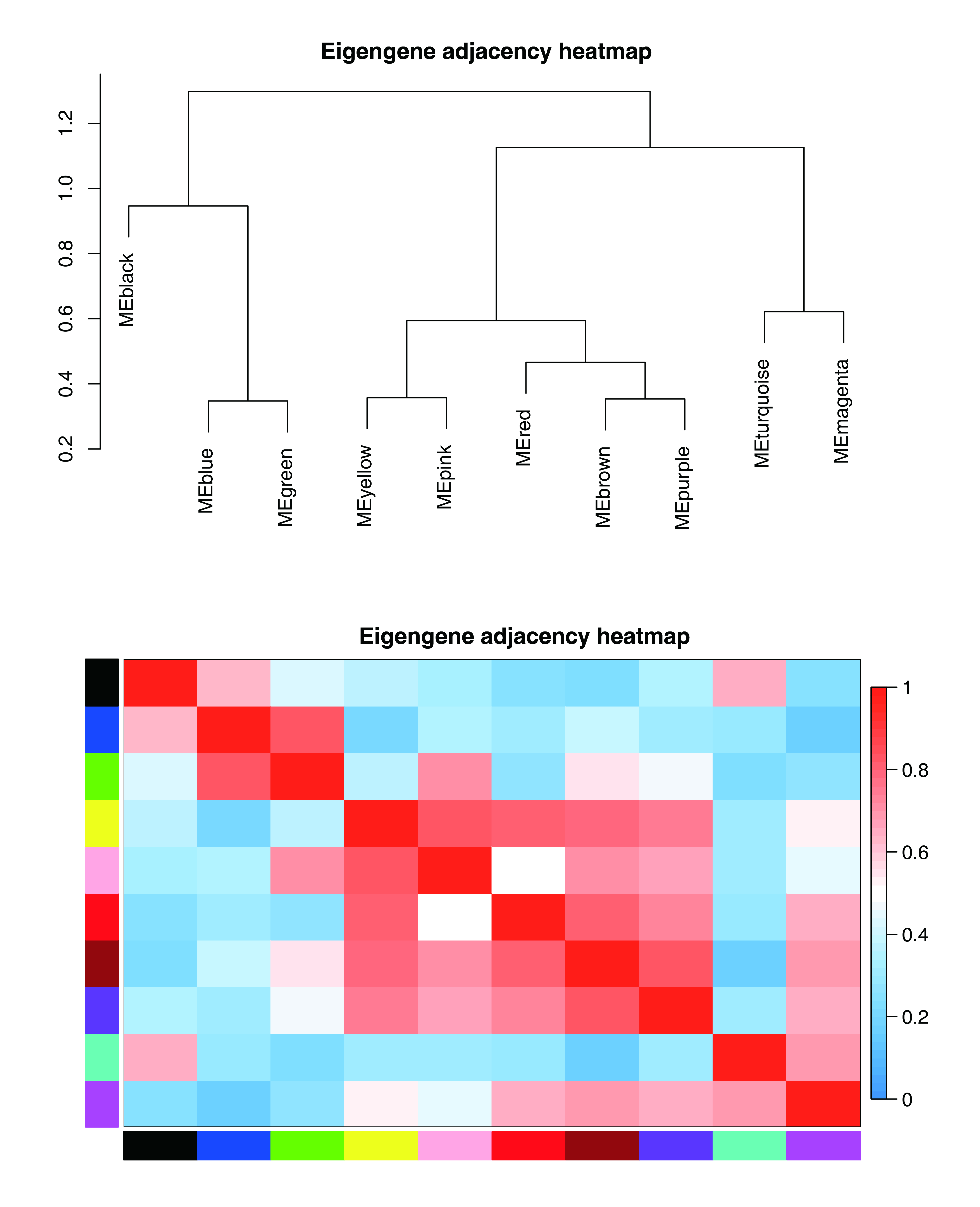

Supplement: Supplementary file 4 [file Image1.tif]
